# By the numbers: ratings and utilization of behavioral health mobile applications

**DOI:** 10.1038/s41746-019-0129-6

**Published:** 2019-06-17

**Authors:** Andrew D. Carlo, Reza Hosseini Ghomi, Brenna N. Renn, Patricia A. Areán

**Affiliations:** 1 Department of Psychiatry & Behavioral Sciences, University of Washington, Seattle, WA USA; 2 Department of Neurology, University of Washington, Seattle, WA USA

**Keywords:** Health services, Public health, Health policy, Health care economics

## Abstract

Although >10,000 behavioral health applications (“apps”) are currently available on the Apple and Google Play marketplaces, they have been minimally evaluated or regulated and little is known about “real world” usage patterns. This investigation combined data from online behavioral health app rating frameworks and a mobile health market research firm to identify the most downloaded apps as well as determine rating and ranking concordance between frameworks. Findings demonstrated that the most commonly downloaded apps focus on relaxation, mindfulness, and meditation skills and that they often have notably discordant reviews across rating frameworks. Our results suggest that there is a growing need for: (1) standardized behavioral health app quality and effectiveness measures, (2) up-to-date behavioral health app guidance for clinicians and consumers, and (3) evidence-based apps that incorporate revealed consumer preferences.

## Introduction

In recent years, the development of mobile behavioral health applications (“apps”) has paralleled the growth in smartphone and digital technologies. The latest estimates suggest that between 165,000 and 325,000 health and wellness apps^1–4^ are now commercially available to patients, with >10,000 designed specifically for mental or behavioral health.^5^ Although a relatively small number of these behavioral health apps have undergone rigorous evaluation in controlled trials,^6–8^ the vast majority remain largely unevaluated^9^ or claim to be evidence-based primarily because they are informed by evidence-based treatments (e.g., cognitive behavioral therapy or mindfulness).^5^ Further, few studies have substantiated the effectiveness of behavioral health apps outside of research settings, limiting the external validity of existing empirical findings.^7,10^ Even when apps are evidence based, their public health impact is often curbed by poor adherence^11–15^ and a lack of availability to the general public through commonly used channels, such as the Apple and Google Play marketplaces.^16,17^

Nevertheless, interest in digital health technologies continues to grow and more than half of mobile device users have downloaded at least one health-related app^18^ at some point in their lives. Owing to the lack of publicly available information on the quality or effectiveness of these apps, purchasing and downloading decisions are often made using heuristics that compel the user to quickly weigh easy-to-identify metrics or attributes, such as title, logo, price, and marketplace star ratings.^19^ To allow consumers to make more informed choices, a number of systematic frameworks (with and without expert reviews^2,20–22^) have been created to rate or rank health apps for different medical conditions (e.g., behavioral health disorders, sickle-cell disease, heart disease, diabetes, and asthma^23^) across a variety of dimensions, including security/privacy, evidence base, ease of use, and interoperability.^24^ However, to date, there is no universally accepted resource or method,^25^ and it remains unclear whether existing tools reach concordance on commonly rated applications.

For years, government oversight and regulation have failed to keep pace with mobile health app growth, leaving consumers potentially vulnerable to apps that claim to offer more than they can deliver. This is expected to be partially addressed in the near future with the United States Food and Drug Administration’s (FDA) recently initiated Digital Health Software Precertification (Pre-Cert) Program, which will allow for the evaluation and monitoring of digital health products, including smartphone apps, from pre-market development to post-market performance.^26^ Although a significant regulatory step, this “opt-in” program is not expected to influence the majority of apps or developers, as most will likely continue to offer products to consumers without the involvement of the FDA. Nevertheless, it remains incumbent upon regulators to ensure that the most commonly used apps are providing high-quality and effective services to consumers.

There is a consequent emerging need to better understand “real world” behavioral health app usage. At present, little is known about which apps are most popular among consumers and whether such popularity aligns with app quality. A recent investigation examined several characteristics of common behavioral and medical health apps, including World Health Organization (WHO) digital intervention classification,^27^ marketplace rating, evidence base, developer “medical claims,” and consumer costs, among others.^17^ Although specific apps were not identified by name, the authors noted considerable content heterogeneity, such that they were unable to make objective quality assessments.^17^ This raises questions about contemporary behavioral health app rating frameworks,^2,20–22^ as they often do not overtly acknowledge their inherent methodological subjectively. At the same time, these frameworks and expert reviews may be playing an important role in helping consumers and clinicians navigate the complex, crowded, and poorly studied behavioral health app marketplace. Therefore, it is necessary to understand how consumer-facing frameworks with expert reviews rate commonly used apps and whether they do so consistently and rigorously.

This investigation aims to address current gaps in the literature by: (1) identifying the most downloaded and installed apps for mental and behavioral health disorders and (2) comparing the ratings of the most downloaded and installed apps from three consumer-facing, publicly available, online rating frameworks with expert reviews.

## Results

### Behavioral health app sample

**Fig. 1:**
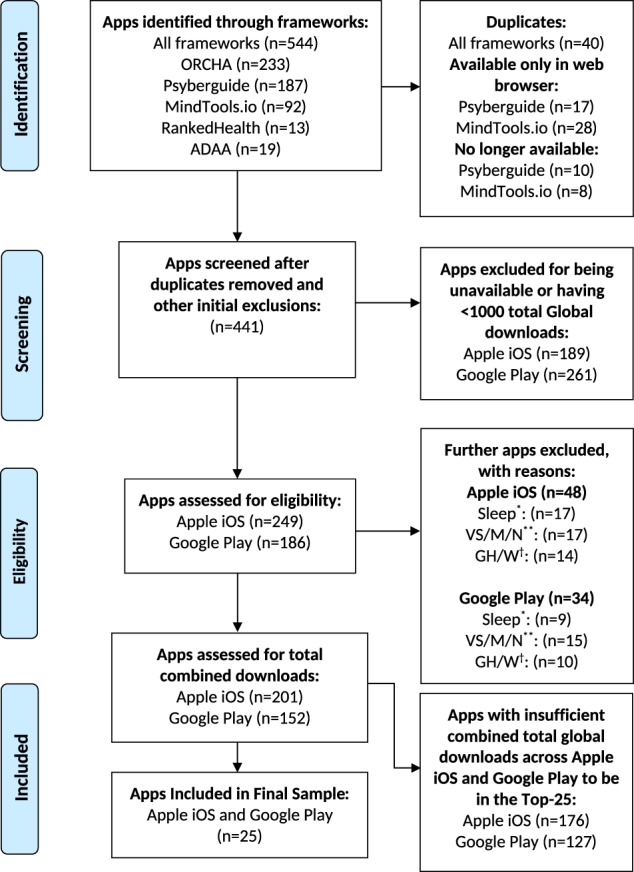
Behavioral health app inclusion and exclusion flow chart. *Sleep apps were excluded if they were not designed to treat a behavioral health disorder or not promoting a specific behavioral health treatment or technique (e.g., mindfulness, meditation). **Apps with a primary focus on nature/soothing visual scenes, music/sounds, or noise/white noise were excluded, although some behavioral health-focused apps did include these features as part of their treatment package. †Apps with a primary focus on general health or wellness were excluded, although some behavioral health-focused apps did include these features as part of their treatment package

A total of 441 unique behavioral health apps remained in the final sample after removing duplicates, discontinued apps, and those only available through a web browser. Apps with <1000 total global downloads since first tracked were excluded from the analysis (189 from Apple and 261 from Google Play). Additionally, 48 Apple and 34 Google Play apps were excluded for having a primary focus on sleep, white noise, nature sounds, or general health/wellbeing). See Fig. 1 for a detailed flow chart on app inclusions and exclusions.

### Downloads and utilization

Table 1 lists the 25 most popular behavioral health apps based on total combined Apple App Store (iOS) and Google Play Store (Android) global downloads. Total global downloads ranged from a high of 42,300,000 for Peak—Brain Training^28^ to 872,600 for Calm Harm.^29^ Overall, apps featuring meditation, mindfulness, and relaxation skills were most common, comprising 19 of the top 25 (76%). The two most downloaded apps, however, were cognitive training applications (Peak—Brain Training^28^ and Lumosity^30^). Three of the top 25 (12%) offered peer support, while 4 (16%) offered mood or anxiety self-monitoring. In addition, one offered virtual psychotherapy (Talkspace^31^), one featured cognitive behavioral therapy (CBT)-inspired games/skills (Happify^32^), one offered support for reducing self-harm behaviors (Calm Harm^29^), and one was a health coach and behavior tracker (Fabulous: Motivate Me^33^). In most cases, total download counts were higher on Apple than on Google Play. Notable exceptions included Lumosity,^30^ Fabulous: Motivate Me,^33^ and Daylio,^34^ all of which had more Google Play downloads.

**Table 1:** Detailed download and utilization data for behavioral health applications (October 2018)

Overall rank	App name	App type	Overall downloads	Apple App Store (iOS)			Google Play Store (Android)		
				TDs^a^	DAUs^b^	MAUs^b^	TDs^a^	DAUs^b^	MAUs^b^
1	Peak—Brain Training	Cognitive training	42M	29M	4M	20M	13M	101K	619K
2	Lumosity	Cognitive training	27M	12M	3M	9M	15M	129K	790K
3	Headspace	Meditation	26M	14M	213K	1M	12M	182K	830K
4	Calm	Meditation	25M	16M	237K	1M	9M	112K	587K
5	Relax Melodies: Sleep Sounds	Meditation	15M	9M	234K	2M	6M	24K	208K
6	Fabulous—Self Care	Health coach, behavior tracker	6M	927K	17K	165K	5M	10K	71K
7	Daylio	Mood tracker	6M	632K	11K	103K	5M	18K	158K
8	Insight Timer	Meditation	5M	3M	51K	409K	2M	8K	64K
9	Stop, Breathe and Think	Meditation	4M	3M	45K	414K	1M	6K	53K
10	Pacifica	Meditation, mood tracking, peer support	3M	3M	95K	838K	738K	2K	18K
11	Simple Habit – Meditation	Meditation, mood tracking, peer support	3M	2M	37K	273K	726K	7K	52K
12	Happify	Cognitive behavioral therapy games, meditation	2M	2M	20K	166K	263K	635	6K
13	7 Cups: Anxiety & Stress Chat	Peer support	2M	2M	31K	278K	749K	2K	21K
14	Breethe - Sleep & Meditation	Meditation	2M	2M	108K	915K	33K	850	8K
15	Smiling Mind	Meditation	2M	2M	45K	426K	392K	3K	35K
16	The Mindfulness App	Meditation	2M	1M	22K	179K	777K	3K	21K
17	Aura: Calm Anxiety & Sleep	Meditation	2M	1M	55K	403K	331K	5K	40K
18	21-Day Meditation Experience	Meditation	2M	1M	13K	102K	389K	616	6K
19	Digipill: Guided Meditation	Meditation	2M	2M	145K	895K	16K	4	86
20	Self-Help for Anxiety Management	Anxiety tracker, meditation	1M	719K	17K	155K	348K	491	5K
21	Take a Break! - Meditations for Stress Relief	Anxiety tracker, meditation	1M	898K	26K	242K	138K	2	27
22	Talkspace	Psychotherapy	985K	835K	13K	113K	150K	539	5K
23	Omvana – Meditation for Everyone	Meditation	964K	910K	10K	92K	55K	64	618
24	Breathe2Relax	Meditation	948K	768K	33K	283K	179K	693	7K
25	Calm Harm	Self-harm reduction	873K	626K	9K	83K	247K	938	9K

*M* million, *K* thousand, *TD* total download, *DAU* daily active user, *MAU* monthly active user

^a^Total download rank was based on sum of Apple App Store (iOS) and Google Play Store (Android) total global downloads since first tracked. Of note, the duration of time tracked by PrioriData varied across individual apps and marketplaces. The same app was often tracked over different periods of time on the Apple and Google Play App Stores

^b^Utilization data, including DAUs and MAUs, describe the 30-day period preceding the PrioriData query

Patterns of monthly (MAU) and daily active user (DAU) counts (Table 1) tended to follow total downloads within the top 25, with cognitive training and meditation apps having the highest utilization. Similar to downloads, utilization figures were higher for Apple than for Google Play. Apple had five apps with more than one million MAUs and two with more than one million DAUs. Google Play had no apps with either of those distinctions. Further, 19 of the top 25 apps had <100,000 Google Play MAUs, while only 2 apps had that few Apple Store MAUs.

### App evaluation frameworks

**Fig. 2:**
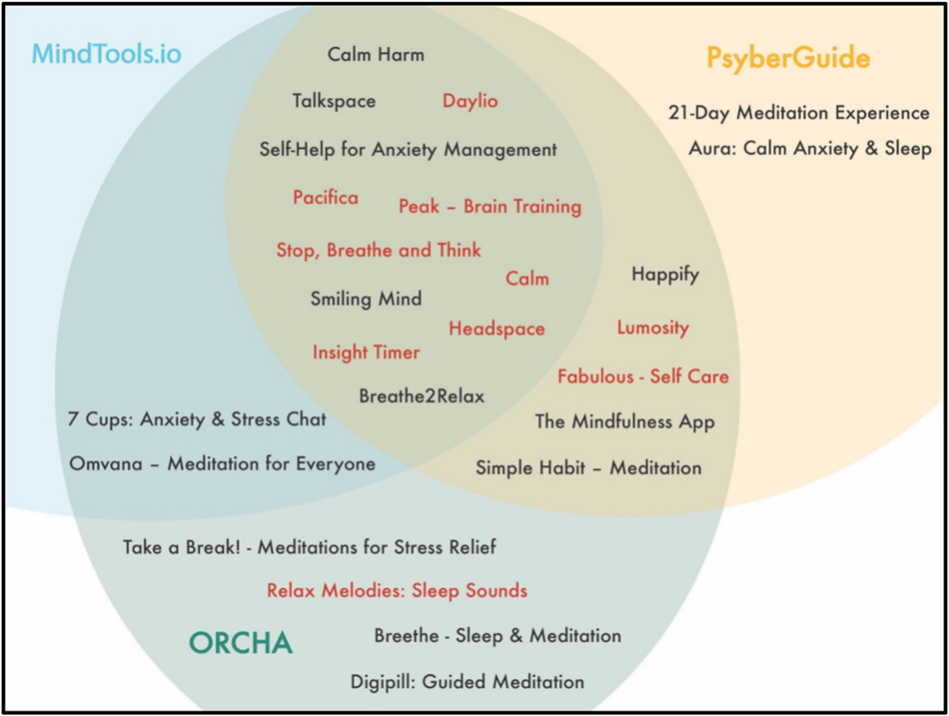
Visualization of framework reach and overlap apps colored in red are in the top ten with regard to total global downloads since first tracked

As detailed in Table 2 for the top 10 apps, there was wide variability in ratings provided by the three evaluation frameworks (see Table S1 in the online supplement for ratings of the top 25 apps). No single framework evaluated all of the most downloaded apps; the Organization for the Review of Care and Health Applications (ORCHA)^35^ reviewed the highest number (*n* = 22) followed by PsyberGuide^36^ (*n* = 19) and MindTools.io^37^ (*n* = 14). There was considerable overlap in app evaluation by the three frameworks (Fig. 2). The mean age (in days) of expert reviews ranged from 109 (ORCHA) to 714 (MindTools.io), while median age (in days) ranged from 38 (ORCHA) to 776 (MindTools.io)—see Table 3 for details. No individual app received top-tercile scores for all categories across all frameworks. Fleiss’ Exact Kappa scores ranged from 0.147 (Data Use & Security) to 0.228 (Credibility & Evidence Base), suggestive of slight to fair reliability overall (Table 4).

**Table 2:** Ratings of the 10 most downloaded behavioral health applications across three expert review evaluation frameworks

Overall download rank^a^	App name	Expert review evaluation framework	Evaluation category		
			User Experience (UE)	Credibility & Evidence Base (CEB)	Data Use & Security (DUS)
1	Peak—Brain Training	MindTools.io	3.2/5 (0.64)^B^	Very good^A^	Transparent^A^
		PsyberGuide	4.52/5 (0.90)^A^	2.86/5 (0.57)^B^	Unacceptable^C^
		ORCHA	26/30 (0.87)^A^	28/40 (0.70)^A^	23.5/30 (0.78)^A^
2	Lumosity	MindTools.io	—	—	—
		PsyberGuide	4.34/5 (0.87)^A^	3.21/5 (0.64)^A^	Acceptable^A^
		ORCHA	26.2/30 (0.87)^A^	35.5/40 (0.89)^A^	21.2/30 (0.71)^A^
3	Headspace	MindTools.io	4.4/5 (0.88)^A^	Very good^A^	Almost transparent^B^
		PsyberGuide	4.74/5 (0.95)^A^	4.64/5 (0.93)^A^	Questionable^B^
		ORCHA	21.2/25 (0.85)^A^	34.9/45 (0.78)^A^	19/30 (0.63)^B^
4	Calm	MindTools.io	3.5/5 (0.70)^A^	Fair^B^	Almost transparent^B^
		PsyberGuide	4.17/5 (0.83)^A^	2.85/5 (0.57)^B^	Questionable^B^
		ORCHA	41.2/50 (0.82)^A^	—	31.4/50 (0.63)^B^
5	Relax Melodies: Sleep Sounds	MindTools.io	—	—	—
		PsyberGuide	—	—	—
		ORCHA	16.5/25 (0.66)^A^	20.2/45 (0.45)^B^	20.6/30 (0.69)^A^
6	Fabulous - Self Care	MindTools.io	—	—	—
		PsyberGuide	—	1.43/5 (0.29)^C^	Unacceptable^C^
		ORCHA	33.1/50 (0.66)^A^	—	21.5/50 (0.43)^C^
7	Daylio	MindTools.io	4/5 (0.80)^A^	Fair^B^	Almost transparent^B^
		PsyberGuide	4.14/5 (0.83)^A^	2.10/5 (0.42)^C^	Questionable^B^
		ORCHA	40.5/50 (0.81)^A^	—	37.2/50 (0.74)^A^
8	Insight Timer	MindTools.io	3.3/5 (0.66)^B^	Good^B^	Not transparent^C^
		PsyberGuide	4.73/5 (0.95)^A^	2.50/5 (0.50)^B^	Unacceptable^C^
		ORCHA	43.7/50 (0.87)^A^	—	27.3/50 (0.55)^B^
9	Stop, Breathe and Think	MindTools.io	3.4/5 (0.68)^B^	Fair^B^	Almost transparent^B^
		PsyberGuide	4.75/5 (0.95)^A^	2.50/5 (0.50)^B^	Unacceptable^C^
		ORCHA	20.2/25 (0.81)^A^	28.7/45 (0.64)^B^	16.1/30 (0.54)^B^
10	Pacifica	MindTools.io	3.3/5 (0.66)^B^	Fair^B^	Almost transparent^B^
		PsyberGuide	4.70/5 (0.94)^A^	2.85/5 (0.57)^B^	Acceptable^A^
		ORCHA	17.6/20 (0.88)^A^	30.6/50 (0.61)^B^	25.1/30 (0.84)^A^

A: top-tercile rating; B: middle-tercile rating; C: bottom-tercile rating

*ORCHA* Organization for the Review of Care and Health Applications

^a^Total download rank was based on sum of Apple App Store (iOS) and Google Play Store (Android) total global downloads since first tracked. Of note, the duration of time tracked by PrioriData varied across individual apps and marketplaces. The same app was often tracked over different periods of time on the Apple and Google Play App Stores

**Table 3:** Framework reach, timeliness, and traffic

		Mindtools.io	Psyberguide	ORCHA
Fraction (%) of top 25 apps reviewed		14/25 (56)	19/25 (76)	22/25 (88)
Website total global visits over 3-month period^a^		22,780	68,041	40,527
Average age of review (days)^b^	Mean	714	598	109
	Median	776	424	38
	Standard deviation	178	305	170

*ORCHA* Organization for the Review of Care and Health Applications

^a^Totals are for the 3-month period between November 2018 and January 2019. Visitors may be counted more than once. Statistics powered by SimilarWeb

^b^Average age of review was calculated as the mean number of days from 15 April 2019

**Table 4:** Rating concordance and tercile distribution of domains across frameworks

User Experience (UE)		Credibility & Evidence Base (CEB)		Data Use & Security (DUS)	
Fleiss’ Exact Kappa (*κ*)^a^—0.13		Fleiss’ Exact Kappa (*κ*)^a^—0.228		Fleiss’ Exact Kappa (*κ*)^a^—0.147	
Fractions of expert reviews by tercile	35/54 (0.65)^A^	Fractions of expert reviews by tercile	12/46 (0.26)^A^	Fractions of expert reviews by tercile	14/54 (0.26)^A^
	13/54 (0.24)^B^		32/46 (0.70)^B^		27/54 (0.50)^B^
	6/54 (0.11)^C^		2/46 (0.043)^C^		13/54 (0.24)^C^

A: top-tercile rating; B: middle-tercile rating; C: bottom-tercile rating

^a^Fleiss’ Kappa (*κ*) is an index of agreement for more than two raters that is adjusted for chance. Interpretation: <0 (poor agreement), 0.0–0.20 (slight agreement), 0.21–0.40 (fair agreement), 0.41–0.60 (moderate agreement), 0.61–0.80 (substantial agreement), 0.81–1.0 (almost perfect agreement)

### User Experience

Across the three frameworks, there were 54 unique expert reviews of User Experience for the top 25 apps. Of the three domains, this had the highest fraction of top (third) tercile ratings (35/54 or 64.8%). The corresponding fractions for the second and first terciles were 13/54 (24.1%) and 6/54 (11.1%), respectively (Table 4).

With a Fleiss’ Exact Kappa score of 0.13 (slight agreement), User Experience ranked at the bottom of the three domains with regard to reliability. It was rated by at least two frameworks for 18 of the apps, with 12 having ratings from all three. Consistency was noted in the top-tercile user experience ratings of Lumosity,^30^ Headspace,^38^ Calm,^39^ Daylio,^34^ and 7 Cups.^40^ Seven of the top 25 apps had zero or one user experience expert review, meaning that we could not assess their degree of concordance. No app had consistently poor ratings across frameworks for this domain. Of note, ratings of user experience seemed to have the most convergence with download ranking, with most apps in the top ten rated favorably in this category.

### Credibility & Evidence Base

Across the three frameworks, there were 46 unique expert reviews of Credibility & Evidence Base for the top 25 apps. The fractions of apps with third- (top), second-, and first-tercile ratings were 12/46 (26.1%), 32/46 (69.6%), and 2/46 (4.3%), respectively (Table 4).

This domain had the highest Fleiss’ Exact Kappa score of the three (0.228—fair agreement). It was rated by at least 2 frameworks for 14 of the apps, with 6 having ratings from all 3. Headspace^38^ was the only app to receive a top-tercile rating for creditability and evidence base by all three frameworks. Six apps had consistent middle-tercile ratings, while no individual app had consistently poor ratings across frameworks for this domain. Eleven of the top 25 apps had zero or one framework rating, meaning that we could not assess their degree of reliability.

### Data Use & Security

Across the three frameworks, there were 54 unique expert reviews of Data Use & Security for the top 25 apps. The fractions of apps with third- (top), second-, and first-tercile ratings were 14/54 (25.9%), 27/54 (50.0%), and 13/54 (24.1%), respectively. Of note, this domain had the largest share of bottom-tercile ratings (Table 4).

Date Use & Security had the middle Fleiss’ Exact Kappa score of the three domains (0.147—slight agreement). It was rated by at least two frameworks for 18 of the apps, with 11 having ratings from all three. None of the apps had universal top-tier ratings, but nine had consistent ratings in the middle- or top-tier (among at least two of the frameworks). Additionally, unique to this domain, four apps had bottom-tier ratings either consistently or in preponderance.

### Independent keyword searches

As described previously, additional PrioriData^41^ keyword searches were conducted for highly downloaded apps meeting study inclusion and exclusion criteria that were not rated by any of the five frameworks (and therefore did not appear in this investigation’s final app sample). This query yielded a total of 40 Apple and 38 Google Play apps that, if included in the final sample for this study, would have had sufficient downloads and installs to be in the 50 most popular in their respective marketplace. These represent estimates of the counts of popular behavioral health apps that were “missed” by using a rating framework-derived sampling strategy.

## Discussion

Our key findings detail the 25 most downloaded behavioral health apps and their corresponding evaluations across online rating frameworks with expert reviews. The issue of how to best evaluate behavioral health apps and disseminate the results is timely and significant. Although this investigation focuses on behavioral health, its methodology and findings are relevant to all of digital health. Psyberguide,^36^ Mintools.io,^37^ and ORCHA^35^ represent three largely transparent attempts to objectively review behavioral health apps across key domains, such as user experience, credibility, evidence base, data privacy, and security.^24^ Our results show that their ratings are broadly inconsistent and often contradictory, with even the most popular behavioral health apps (as determined by total global downloads) not receiving uniformly favorable scores. Quantitatively, we demonstrated that Fleiss’ Exact Kappa scores for the three domains ranged from 0.147 (Data Use & Security) to 0.228 (Credibility & Evidence Base), suggestive of only slight to fair reliability overall. This could, in part, be a consequence of the frameworks’ inclusion of inherently subjective and poorly reliable categories like User Experience,^42^ instead of more general and measurable consumer preferences.^17^ At the same time, our findings also noted significant discordance in more objective categories like Data Use & Security, suggesting that inconsistencies are multifactorial and cannot be ascribed solely to fundamental domain characteristics. Ultimately, inter-framework discrepancies are most likely a consequence of the current lack of consensus domains and standards for behavioral health apps.

Even with such standards, however, it remains an open question whether frameworks could keep mobile health app evaluations up-to-date and effectively disseminate them to the general public longitudinally. Of the three examined frameworks, ORCHA reviewed the largest percentage of the 25 most downloaded apps (88%), indicating that it may be most in line with the public’s revealed preferences (as measured by app downloads). Other frameworks, by this measure, were less up-to-date, with corresponding figures for Psyberguide and Mindtools.io being 76% and 56%, respectively. ORCHA was also the most current for the top 25 apps, with a mean age of expert review of 109 days, less than one-fifth that of the second most current framework, Psyberguide. Given the expected continuation of rapid growth in the behavioral health app field, the challenge of keeping expert reviews up-to-date will only grow more arduous over time, raising questions about the maximum potential impact of online behavioral health app rating frameworks on public knowledge and decision-making. In fact, according to the authors’ SimilarWeb-powered analysis of web traffic data,^43^ the most accessed of the three online frameworks, Psyberguide, was visited by a total of 68,041 users between November 2018 and January 2019. When this is juxtaposed against Peak—Brain Training’s 42 million downloads, >300,000 Google Play reviews, and >85,000 Apple reviews, it appears that many customers may be seeking guidance from sources outside of the rating frameworks.

Although this does not discount the potential benefits of having objective, expert reviewers for behavioral health apps, it suggests that other strategies, such as data crowd sourcing or citizen science,^44^ may be more scalable, effective, and sustainable. It also suggests that frameworks designed to facilitate shared app decision-making, such as the American Psychiatric Association’s (APA) app evaluation model^45^ and others,^2,23^ may be valuable tools for consumers, clinicians, and patients without providing ongoing expert reviews.

Despite notable limitations, there is valuable information to be gleaned from the app rating and ranking frameworks in this investigation, all of which have attempted to organize and present complex information in a succinct and comprehensible manner to the general public. This is particularly true in cases of concurrence across frameworks. For example, irrespective of framework, only 26% (12/46) of the ratings of Credibility & Evidence Base for the top 25 apps were top-tercile. This finding is alarming, but also unsurprising, given the well-described lack of rigorous research on behavioral health and other health-related apps.^46^ It does not, however, appear to stop developers from marketing apps as either standalone or adjunctive treatments for common behavioral health disorders (e.g., depression, anxiety); let the buyer beware of such tools in the absence of universal standards. Similarly, limited transparency around Data Use & Security led 24% (13/54) of the ratings to be bottom-tercile, the highest percentage of the three domains. The need for data privacy in the digital age is paramount, yet a lack of transparency and standardization remains prevalent in the marketplace.^47^

In addition to demonstrating that popular behavioral health apps may have poor or questionable support from the literature, the results of this investigation also showed that the most commonly downloaded apps (and also those with some of the highest counts of DAUs and MAUs) simply offered relaxation, meditation, or mindfulness skills, rather than bona fide behavioral health treatments. These frequently downloaded and used apps have defied the odds, as research has demonstrated that many users stop accessing behavioral health digital interventions within 2 weeks of the initial download.^48^ This poses an important question—what role do consumers feel that mobile technology should play in behavioral health recovery? As a recently published survey uncovered, most people who would consider using app-based care interventions were skeptical of completely self-guided tools.^49^ This could signify that behavioral health apps are most suitable for tracking or mindfulness and that evidence-based treatments (e.g., psychotherapy or brief interventions) are best reserved for traditional care. It also suggests that academic researchers and clinicians designing and evaluating apps may be missing what consumers are actually seeking. If researchers do aim to make mobile health interventions with evidence-based treatments attractive and accessible in the “real world,” they should be sure to focus on subjective constructs (e.g., user experience) early in the design phase and ensure that their apps are available on the Apple and Google Play marketplaces. Owing to the requirement of diverse skillsets and expertise, it is likely that successful promotions of evidence-based behavioral health apps will require ongoing, meaningful collaborations between clinicians, thought leaders, and digital user experience professionals.

Although rigorous sampling methods were employed in this investigation, our final sample was limited by the content of the five included frameworks. This largely restricted our focus to English language apps, despite our lack of specific exclusion criteria for apps unavailable in English. Our finding of additional Apple (iOS) and Google Play (Android) apps through PrioriData^41^ keyword searches demonstrated that a number of popular apps were “missed” using this strategy. The “missing” apps were either unrated by popular rating frameworks for unknown reasons or had not yet been rated at the time of the study. The use of a framework-derived sampling strategy, however, allowed the authors to assess the degree of concordance between highly visible, consumer-facing frameworks, which was a primary objective of this investigation. Further, our sampling design forced exclusion of apps that were either (1) available only through a web browser or (2) unavailable on the Apple or Google Play marketplaces. This was necessary because PrioirData^41^ could not provide download or utilization data on apps that were not available through one of the two primary marketplaces. Of note, it is almost certain that any sampling technique would have missed large numbers of apps, given the >10,000 that are thought to be available for behavioral health. Additionally, this investigation was limited by the constraints of our data source. Although PrioriData^41^ uses rigorous methodology to obtain its estimates of total downloads, DAUs, and MAUs, these results are largely model derived and are subject to statistical error margins (which were not available to the authors for this investigation). Finally, for total downloads, all behavioral health apps were tracked for different periods of time on PrioriData.^41^ Identical apps on Apple and Google Play were also tracked for different periods of time, depending on when the app was made available by the developer on the particular marketplace. However, the primary purpose of this investigation was to provide an estimate of total counts of unique downloads in the “real world,” as opposed to providing a download rate over time.

Although the behavioral health app market has grown rapidly in recent years, it has been minimally evaluated or regulated and little is known about “real world” usage patterns. The results of this investigation demonstrate that the most commonly downloaded and installed behavioral health apps tend to focus on relaxation, mindfulness, and meditation, as opposed to bona fide treatments. In addition, findings suggest that consumer-facing behavioral health app rating and ranking frameworks fail to score a number of popular apps and are often discordant in their existing evaluations. Finally, there is a clear gap between behavioral health research and consumers’ revealed preferences. Successful promotions of evidence-based apps will likely require ongoing, meaningful collaborations between clinicians, thought leaders, and digital user experience professionals.

## Methods

### Behavioral health app sample

The authors obtained the study sample of mobile (smartphone- or tablet-based) behavioral health apps from five consumer-facing, publicly available online rating frameworks with expert reviews^35–37,50,51^ identified in recently published literature.^5,23,52^ One framework (AppScript^53^) was excluded, as it was primarily designed for health providers (e.g., physicians, nurses, health coaches) instead of patients or general consumers. Three of the five included frameworks rated only behavioral health apps,^36,37,51^ while the other two rated health apps of all types.^35,50^ All apps from the three exclusively behavioral health frameworks (Psyberguide,^36^ Mindtools.io^37^ and the Anxiety and Depression Association of America (ADAA)^51^) were included in the study sample. For ORCHA,^35^ all apps flagged from keyword searches for “Stress and Anxiety” and “Depression” were included. For RankedHealth,^50^ all apps were manually reviewed and included if there was any clear, broadly defined association with mental or behavioral health. Duplicate apps were removed from the final study sample.

Apps were excluded from the final sample if they were: (1) functional only through a web browser interface (because our market research firm’s (PrioriData^41^) download/installation and utilization estimates are partially derived from mobile app marketplaces), (2) no longer on the market, and (3) not designed to treat a behavioral health disorder or not promoting a specific behavioral health treatment or technique (e.g., mindfulness, meditation). To exemplify this third criterion, apps for sleep were included only if they included a specific behavioral health treatment or technique, such as CBT for insomnia.^54^ This excluded apps that promoted sleep through digital alarm clocks, “trackers,” or other methods. In addition, apps whose primary focus was not a behavioral health technique (e.g., imagery apps, white noise apps, or health and wellness apps) were excluded, although some behavioral health-focused apps did include these features as part of their treatment packages. Decisions to include/exclude apps were made independently by two members of the research team (A.D.C., R.H.G., or B.N.R.). Discrepant decisions were discussed during team meetings and revisions were made accordingly.

### Market research data—PrioriData^41^

Download, installation, and utilization data for all behavioral health apps were procured from PrioriData,^41^ a leading mobile app market research firm based in Berlin, Germany that has been cited in previous literature.^55–57^ PrioriData^41^ uses publicly available data, proprietary data (from strategic partners), and modeling techniques to estimate download and utilization rates for mobile apps worldwide.^41^ Each day, PrioriData^41^ obtains actual event, demographic, location, device, and installed app data from >3 billion unique-user devices across >100 countries.^41^ When applicable, data obtained for this investigation were available for all apps from the Apple and Google Play Stores, the two leading marketplaces worldwide. The authors had access to PrioriData’s^41^ platform between 21 September and 21 October 2018. All download and utilization data for this investigation were obtained over the course of this 30-day period.

Separately for the Apple and Google Play marketplaces, PrioriData^41^ provided the following global estimates for all included study apps: (1) total downloads since first tracked, (2) DAUs from the past 30 days, and (3) MAUs from the past 30 days. Of note, for total global downloads, all apps were not tracked for the same amount of time. The “first tracked” date corresponds to the time point at which PrioriData^41^ was able to detect and estimate data for an app. This time point typically occurs shortly after an app is available on the Apple or Google Play marketplace. Finally, the authors ran app keyword searches for the following eight terms—“Depression,” “Anxiety,” “Mood Disorder,” “Mental Health,” “Behavioral Health,” “Psychiatry,” “Psychology,” and “Stress.” Each was queried for total downloads, DAUs, and MAUs. The top 500 results from each query were downloaded. The intention of these keyword searches was to identify behavioral health apps that were not rated in any of the five aforementioned frameworks.

### Association of download, installation, and utilization data with framework ratings

After data were procured for all apps and appropriate exclusions were made from the sample, the authors summed the total Apple and Google Play global downloads (since first tracked) for all apps. For the top 25 apps by combined total downloads, ratings were compared across the Psyberguide,^36^ Mintools.io,^37^ and ORCHA^35^ frameworks. These three frameworks were chosen because they each rated a substantial number of behavioral health apps and are public-facing tools meant to appeal to consumers, clinicians, and researchers. Each framework was different in its structure, methodology, and terminology, with details available online. Psyberguide^36^ (https://psyberguide.org/about-psyberguide/) and Mintools.io^37^ (https://mindtools.io/mindtools-io-scoring/) each consistently had three rating categories, while ORCHA^35^ (https://www.orcha.co.uk/our-solution/the-orcha-review/#0) varied between having three and four. The authors did not include or consider ORCHA’s^35^ fourth category (overall score), as Psyberguide^36^ and Mintools.io^37^ did not have a corresponding category. Although each framework had its own category labels, they all assessed analogous app characteristics and constructs. This made it possible for us to group similar categories together for ease of comparison across frameworks. Program Quality (MindTools.io^37^) and User Experience (PsyberGuide,^36^ ORCHA^35^) were grouped under the “User Experience” category. Source Credibility (MindTools.io^37^), Credibility (PsyberGuide^36^), and Clinical Assurance (ORCHA^35^) were grouped under “Credibility & Evidence Base.” Finally, Privacy Explanation (MindTools.io^37^), Transparency (PsyberGuide^36^), and Data Security (ORCHA^35^) were grouped as “Data Use & Security.”

The three frameworks also differed in the presentation of their data, with some electing to provide numeric or fractional scores and others using qualitative scores. Even within categories of the same framework, data presentation varied significantly at times. For example, within ORCHA^35^ rating categories, fractional scores were occasionally presented with different denominators. Whenever possible, fractional scores were converted to decimals for consistency and ease of comparison.

Terciles were determined differently for each framework and metric. In its detailed methodology, Mindtools.io^37^ provides a key for interpretation of each score. This was used to determine terciles for all Mindtools.io^37^ categories. Of note, in categories with more than three score levels (either quantitative or qualitative), terciles were not able to be matched one-to-one. ORCHA^35^ publishes three-level interpretative ranges (green, yellow, and red) for ratings that are based on the percentage score for each category or domain. To best maintain the integrity of the ORCHA^35^ rating system, these score ranges were maintained and directly used to inform tercile assignment (i.e., green scores from ORCHA were considered top-tercile in this study). In cases where ORCHA provided more than one evaluation of the same app (e.g., if more than one release was evaluated and both remained visible on the website), the newest app version or latest review was used. Since Psyberguide^36^ does not provide clear interpretive guidance for comparison of its composite quantitative scores, tercile ranges were calculated from the distribution of all available scores and each app was assigned accordingly. For Psyberguide’s^36^ three-level qualitative metric (transparency), terciles were matched one-to-one. To assess the reliability of agreement between frameworks, the authors calculated Fleiss’ Exact Kappa for each individual rating domain. Fleiss’ Exact Kappa (*κ*) is an index of agreement for more than two raters that is adjusted for chance.^58^ Although there is no universal interpretive scale, the following is frequency used: <0 (poor agreement), 0.0–0.20 (slight agreement), 0.21–0.40 (fair agreement), 0.41–0.60 (moderate agreement), 0.61–0.80 (substantial agreement), and 0.81–1.0 (almost perfect agreement).^59^ All calculations were conducted using Microsoft Excel and R’s “irr” package.^60^ Additionally, the authors used SimilarWeb^43^ to analyze web traffic data (including total users) for all three frameworks between November 2018 and January 2019.

Finally, the authors calculated how commonly an app appeared across frameworks and how up-to-date each framework was (timeliness). We calculated the percentage of top 25 apps that were rated by each framework, and the mean difference (in days) between the most recent online expert review for each top 25 app and 15 April 2019. Of note, Mindtools.io,^37^ Psyberguide,^36^ and ORCHA^35^ publish their date of review, while RankedHealth^50^ and ADAA^51^ do not. For frameworks that provided month and year only for expert reviews, the date of review was assumed to be the 15th day of the month.

This protocol was reviewed and granted exemption by the University of Washington Human Subjects Division (Protocol #: 5714).

### Reporting summary

Further information on research design is available in the Nature Research Reporting Summary linked to this article.

## Supplementary information

Supplement

Reporting Summary

## Data Availability

The data generated during and/or analyzed in this study are available from adc42@uw.edu on reasonable request.

## Supplementary information

**Supplementary information** accompanies the paper on the *npj Digital Medicine* website (10.1038/s41746-019-0129-6).
