# Supplementary material for: By the numbers: ratings and utilization of behavioral health mobile applications
Source: NPJ Digit Med. 2019 Jun 17;2:54. doi: 10.1038/s41746-019-0129-6 (PMC6572775; doi:10.1038/s41746-019-0129-6)
Supplement: Supplementary file 1 — Supplement [file 41746_2019_129_MOESM1_ESM.pdf]

Table S1: Ratings of the 25 Most Downloaded Behavioral Health Applications Across Three Expert Review Evaluation Frameworks					
Overall Download Rank*	App Name	Expert Review Evaluation Framework	Evaluation Category		
			User Experience (UE)	Credibility & Evidence Base (CEB)	Data Use & Security (DUS)
1	Peak – Brain Training	MindTools.io	3.2/5 (0.64)[b]	Very Good[a]	Transparent[a]
		PsyberGuide	4.52/5 (0.90)[a]	2.86/5 (0.57)[b]	Unacceptable[c]
		ORCHA**	26/30 (0.87)[a]	28/40 (0.70)[a]	23.5/30 (0.78)[a]
2	Lumosity	MindTools.io	-	-	-
		PsyberGuide	4.34/5 (0.87)[a]	3.21/5 (0.64)[a]	Acceptable[a]
		ORCHA**	26.2/30 (0.87)[a]	35.5/40 (0.89)[a]	21.2/30 (0.71)[a]
3	Headspace	MindTools.io	4.4/5 (0.88)[a]	Very Good[a]	Almost Transparent[b]
		PsyberGuide	4.74/5 (0.95)[a]	4.64/5 (0.93)[a]	Questionable[b]
		ORCHA**	21.2/25 (0.85)[a]	34.9/45 (0.78)[a]	19/30 (0.63)[b]
4	Calm	MindTools.io	3.5/5 (0.70)[a]	Fair[b]	Almost Transparent[b]
		PsyberGuide	4.17/5 (0.83)[a]	2.85/5 (0.57)[b]	Questionable[b]
		ORCHA**	41.2/50 (0.82)[a]	-	31.4/50 (0.63)[b]
5	Relax Melodies: Sleep Sounds	MindTools.io	-	-	-
		PsyberGuide	-	-	-
		ORCHA**	16.5/25 (0.66)[a]	20.2/45 (0.45)[b]	20.6/30 (0.69)[a]
6	Fabulous - Self Care	MindTools.io	-	-	-
		PsyberGuide	-	1.43/5 (0.29)[c]	Unacceptable[c]
		ORCHA**	33.1/50 (0.66)[a]	-	21.5/50 (0.43)[c]
7	Daylio	MindTools.io	4/5 (0.80)[a]	Fair[b]	Almost Transparent[b]
		PsyberGuide	4.14/5 (0.83)[a]	2.10/5 (0.42)[c]	Questionable[b]
		ORCHA**	40.5/50 (0.81)[a]	-	37.2/50 (0.74)[a]
8	Insight Timer	MindTools.io	3.3/5 (0.66)[b]	Good[b]	Not Transparent[c]
		PsyberGuide	4.73/5 (0.95)[a]	2.50/5 (0.50)[b]	Unacceptable[c]
		ORCHA**	43.7/50 (0.87)[a]	-	27.3/50 (0.55)[b]
9	Stop, Breathe and Think	MindTools.io	3.4/5 (0.68)[b]	Fair[b]	Almost Transparent[b]
		PsyberGuide	4.75/5 (0.95)[a]	2.50/5 (0.50)[b]	Unacceptable[c]
		ORCHA**	20.2/25 (0.81)[a]	28.7/45 (0.64)[b]	16.1/30 (0.54)[b]
10	Pacifica	MindTools.io	3.3/5 (0.66)[b]	Fair[b]	Almost Transparent[b]
		PsyberGuide	4.70/5 (0.94)[a]	2.85/5 (0.57)[b]	Acceptable[a]
		ORCHA**	17.6/20 (0.88)[a]	30.6/50 (0.61)[b]	25.1/30 (0.84)[a]
11	Simple Habit – Meditation	MindTools.io	-	-	-
		PsyberGuide	3.51/5 (0.70)[b]	2.50/5 (0.50)[b]	Acceptable[a]
		ORCHA**	41.7/50 (0.83)[a]	-	31/50 (0.62)[b]
12	Happify	MindTools.io	-	-	-
		PsyberGuide	3.34/5 (0.67)[c]	3.92/5 (0.78)[a]	Questionable[b]
		ORCHA**	22/25 (0.88)[a]	36.5/45 (0.81)[a]	23.8/30 (0.79)[a]
13	7 Cups: Anxiety &	MindTools.io	3.8/5 (0.76)[a]	Fair[b]	Almost Transparent[b]

	Stress Chat	PsyberGuide	-	-	-
		ORCHA**	18.1/20 (0.91)[a]	45/50 (0.90)[a]	19.6/30 (0.65)[a]
14	Breethe - Sleep & Meditation	MindTools.io	-	-	-
		PsyberGuide	-	-	-
		ORCHA**	32.3/50 (0.65)[b]	-	24.8/50 (0.50)[b]
15	Smiling Mind	MindTools.io	3.1/5 (0.62)[b]	Good[b]	Almost Transparent[b]
		PsyberGuide	4.00/5 (0.80)[a]	2.85/5 (0.57)[b]	Unacceptable[c]
		ORCHA**	23.8/30 (0.79)[a]	22.2/40 (0.56)[b]	15.6/30 (0.52)[b]
16	The Mindfulness App	MindTools.io	-	-	-
		PsyberGuide	3.30/5 (0.66)[c]	2.85/5 (0.57)[b]	-
		ORCHA**	16.2/25 (0.65)[b]	24.2/45 (0.54)[b]	15.1/30 (0.50)[b]
17	Aura: Calm Anxiety & Sleep	MindTools.io	-	-	-
		PsyberGuide	-	2.86/5 (0.57)[b]	Unacceptable[c]
		ORCHA**	-	-	-
18	21-Day Meditation Experience	MindTools.io	-	-	-
		PsyberGuide	3.13/5 (0.63)[c]	2.85/5 (0.57)[b]	Questionable[b]
		ORCHA**	-	-	-
19	Digipill: Guided Meditation	MindTools.io	-	-	-
		PsyberGuide	-	-	-
		ORCHA**	19.6/25 (0.78)[a]	32.9/45 (0.73)[a]	14.4/30 (0.48)[b]
20	Self-Help for Anxiety Management	MindTools.io	2.7/5 (0.54)[c]	Fair[b]	Not Transparent[c]
		PsyberGuide	4.10/5 (0.82)[a]	2.50/5 (0.50)[b]	Questionable[b]
		ORCHA**	42.8/50 (0.86)[a]	-	35.3/50 (0.71)[a]
21	Take a Break! - Meditations for Stress Relief	MindTools.io	-	-	-
		PsyberGuide	-	-	-
		ORCHA**	29.5/50 (0.59)[b]	-	Numeric value not provided[a]
22	Talkspace	MindTools.io	4.2/5 (0.84)[a]	Good[b]	Almost Transparent[b]
		PsyberGuide	3.57/5 (0.71)[b]	3.57/5 (0.71)[a]	Acceptable[a]
		ORCHA**	16.8/20 (0.84)[a]	31.2/50 (0.62)[b]	19.3/30 (0.64)[b]
23	Omvana – Meditation for Everyone	MindTools.io	2.5/5 (0.50)[c]	Fair[b]	Not Transparent[c]
		PsyberGuide	-	-	-
		ORCHA**	30.8/50 (0.62)[b]	-	22/50 (0.44)[c]
24	Breathe2Relax	MindTools.io	2.5/5 (0.50)[c]	Good[b]	Not Transparent[c]
		PsyberGuide	3.98/5 (0.80)[a]	2.85/5 (0.57)[b]	-
		ORCHA**	39.2/50 (0.78)[a]	-	13.2/50 (0.26)[c]
25	Calm Harm	MindTools.io	3.4/5 (0.68)[b]	Fair[b]	Almost Transparent[b]
		PsyberGuide	4.28/5 (0.86)[a]	2.86/5 (0.57)[b]	Questionable[b]
		ORCHA**	12.6/20 (0.63)[b]	23.5/50 (0.47)[b]	16.2/30 (0.54)[b]

a: top-tercile rating; b: middle-tercile rating; c: bottom-tercile rating

*Total Download Rank was based on sum of Apple App Store (iOS) and Google Play Store (Android) total global downloads since first tracked. Of note, the duration of time tracked by PrioriData varied across individual apps and marketplaces. The same app was often tracked over different periods of time on the Apple and Google Play App Stores.

**ORCHA - Organization for the Review of Care and Health Applications
